# Knowledge and awareness about STDs among women in Bangladesh

**DOI:** 10.1186/1471-2458-14-775

**Published:** 2014-07-31

**Authors:** Mosharaf Hossain, Kulanthayan KC Mani, Sherina Mohd Sidik, Hayati Kadir Shahar, Rafiqul Islam

**Affiliations:** Department of Community Health, Faculty of Medicine and Health Science, University Putra Malaysia, 43400 UPM Serdang, Selangor Malaysia; Department of Psychiatry, Faculty of Medicine and Health Science, University Putra Malaysia, 43400 UPM Serdang, Selangor, Malaysia; Department of Population Science and Human Resource Development, University of Rajshahi, Rajshahi, 6205 Bangladesh

**Keywords:** STDs, Knowledge, Awareness, HIV/AIDS, Women

## Abstract

**Background:**

Knowledge and awareness concerning sexually transmitted diseases (STDs) has become the burning issue of the day. Although STDs pose serious risks to health security, there is very little literature quantifying the knowledge and awareness of these diseases and their principal socioeconomic determinants. The aim of this study is to determine the effect of different socio-economic and demographic factors on knowledge and awareness about STDs among women in Bangladesh.

**Methods:**

This is a cross-sectional study using data from the Bangladesh Demographic and Health Survey (BDHS) 2011. It involves 10,996 women in six divisions of Bangladesh – Dhaka, Rajshahi, Chittagong, Barisal, Khulna and Sylhet. In this study, the percentage distribution and logistic regression model are used to identify which factors are associated with knowledge and awareness among women in Bangladesh about STDs.

**Results:**

There is a significant association between geographic division (Dhaka: OR = 1.669, 95% CI = 0.89-2.10, Khulna: OR = 2.234, 95% CI = 1.2-3.2); places of residence (Rural: OR = 0.363, 95% CI = 0.20-1.08), respondent’s age (20-29 years: OR = 1.331; 95% CI = 0.98-2.31); education (Primary: OR = 2.366, 95% CI = 1.98-3.1, secondary: OR = 10.089, 95% CI = 8.98-12.77, higher: OR = 20.241, 95% CI = 18.33-22.65); listening to radio (OR = 1.189, 95% CI = 1.29-3.12) and watching TV (OR = 2.498, 95% CI = 2.22-4.09) with knowledge and awareness among women in Bangladesh about STDs.

**Conclusion:**

There is a need to improve the education in Bangladesh about STDs particularly among those in the rural areas and older ages of women (30-49 years). Formal, informal and special educational knowledge and awareness programmes may be implemented to educate people concerning STDs in Rajshahi, Sylhet and Chittangong division. Campaigns and mass media can be used to increase the knowledge and awareness among the community, especially among women. Policies concerning the issue of STDs need to be improved and can be emphasized in collaboration with government agencies to ensure the success of these campaigns.

## Background

Bangladeshi women are very vulnerable to STDs, including HIV/AIDS, and their knowledge about different diseases is extremely poor. Sexually transmitted diseases (STDs) increase the likelihood of HIV transmission as well as other reproductive health consequences, such as chronic lower abdominal pain, infertility or life threatening pregnancies [1]. The World Health Organization (WHO) estimates that at least one third of the 333 million new cases each year of curable sexually transmitted infections (STIs) occur among people under 25 years of age [2]. It was estimated that, at the end of 2001, approximately 40 million people worldwide were living with HIV/AIDS, of which 6.4 million people belonged to the Asian region. Young people bear a special burden in the HIV/AIDS pandemic, and adolescent women are particularly vulnerable to sexually transmitted diseases and HIV/AIDS [3]. Among adolescents, girls are more vulnerable to STDs than their male counterparts, including HIV/AIDS, especially through heterosexual intercourse with others. This increased vulnerability is attributable to issues beyond their control, such as sexual violence and exploitation, early sexual initiation and the inability to negotiate for safe sex. Other contributing factors include strong discrimination, lack of education, lack of power, lack of access to contraception and reproductive health issues. Therefore, it is difficult for adolescent women to protect themselves from sexually transmitted diseases, HIV and unwanted pregnancies. In addition, young people are not informed about the sexually transmitted diseases, and their knowledge about the different diseases is very poor. Although there have been many studies that have examined the knowledge about STDs among various age groups and social groups, very few studies have investigated the level of knowledge of young women about STDs. The knowledge of women concerning either the mode of transmission or prevention of STDs is very limited [4]. An integrated approach including useful and fruitful media campaigns to educate woman about the health consequences of STDs including HIV/AIDS is strongly suggested for creating knowledge and awareness, and for controlling the spread of STDs among young women in Bangladesh [5].

Bangladesh is a low HIV/AIDS prevalent country but it is at a critical moment in the course of its AIDS epidemic [6–9]. According to the UNAIDS estimates, Bangladesh, with a population of 136 million, had about 13,000 people living with HIV/AIDS at the end of 2001, with the HIV prevalence in the adult population being less than 0.01% [9]. However, although the overall prevalence of HIV in Bangladesh is low, it is a high-risk country for HIV/AIDS. This is due to the presence of covert multi-partner sexual activity, the low level of knowledge and low condom use, unsafe professional blood donation, high incidence of self-reported sexually transmitted infections among vulnerable groups, the return of expatriates working in different countries, and the high levels of HIV/AIDS in the two neighbouring countries, India and Myanmar [6–10]. The country’s vulnerability is very high compared to other parts of South Asia, and infection rates within the vulnerable groups are increasing, thereby leading to an ever-greater possibility that the virus will spread to the general population [6, 7, 11]. In this critical situation, public awareness can play a dominant role in preventing an epidemic of HIV/AIDS [12]. However, the awareness level and knowledge of the correct ways to avoid HIV/AIDS among the general public in Bangladesh are quite low. Among the men aged 15–54, 18% have never heard of HIV/AIDS, 24% have heard but do not know of any correct way to avoid it and only 58% know one or more correct ways to avoid the disease [12]. On the other hand, 40% of ever-married women have never heard of HIV/AIDS, 19% have heard but do not know any correct way and only 41% know one or more correct ways to avoid the disease [12]. These situations have raised serious concerns among the government and the various stakeholders and they are seeking to increase the public awareness concerning the transmission and prevention of HIV. Therefore, it is important to identify the reasons that are associated with the level of awareness, which will be helpful in strengthening the capacity of government/non-government organizations (NGO)/development-partner agencies for programme planning, implementation, monitoring and evaluation regarding AIDS awareness. In this regard, a few national and international researchers have attempted to understand the reasons and offer some explanations [10, 12, 13]. The respondents’ education level, contraceptive usage, mass media and HIV workshops was statistically positive significant effects on HIV/AIDS knowledge and the awareness of female garment workers [14]. HIV/AIDS is preventable; only 45% of respondents said HIV/AIDS is not curable and 70.5% answered that death is the ultimate fate. The respondent’s main source of information concerning HIV/AIDS is taken from the radio/TV and newspapers. Unfortunately, 76.9% of the respondents have poor knowledge and awareness about HIV/AIDS while only 10.6% have good knowledge and awareness. The level of knowledge and awareness of HIV/AIDS decreases with an increase in age (p < 0.05). Although the males are slightly more aware than the females, the relationship is not statistically significant (p > 0.05). Secondary educated female garment workers have more knowledge and awareness about HIV/AIDS, including STDs, than illiterate female garment workers (p < 0.01) [15]. Therefore, the aim of this study is to determine the role of different socio-economic and demographic factors concerning the knowledge and awareness among the women in Bangladesh about STDs.

## Methods

### Sample design

The sample for the 2011 Bangladesh Demographic and Health Survey (BDHS) was nationally representative and covered the entire population not residing in institutional dwelling units in the country. The survey was used as a sampling frame for the list of enumeration areas (EAs) prepared for the 2011 Population and Housing Census, provided by the Bangladesh Bureau of Statistics (BBS). The primary sampling unit (PSU) for the survey was an EA that was created to have an average of about 120 households. Bangladesh has six administrative divisions: Barisal, Chittagong, Dhaka, Khulna, Rajshahi, and Sylhet. Each division is subdivided into *zilas (meaning district)*, and each *zila* into *upazilas (meaning administrative unit)*. Each urban area in an *upazila* is divided into wards, and into *mohallas (meaning city corporation unit)* within a ward. A rural area in the *upazila* is divided into *union parishads* (UP) and *mouzas (meaning village)* within a UP. These divisions allow the country as a whole to be easily separated into rural and urban areas. The survey was based on a two-stage stratified sample of households. In the first stage, 600 EAs were selected with the probability proportional to the EA size, with 207 clusters in urban areas and 393 in rural areas. A complete household listing operation was then carried out in all the selected EAs to provide a sampling frame for the second-stage selection of households. In the second stage of sampling, a systematic sample of 30 households, on average, was selected per EA to provide statistically reliable estimates of key demographic and health variables for the country as a whole, for urban and rural areas, separately, and for each of the six divisions. With this design, the survey selected 10,996 residential households, which were expected to result in completed interviews with about 10,996 ever-married women. The sample allocations were derived using information obtained from the 2007 BDHS. Based on the 2007 data, the average number of ever-married women age 12-49 per household was assumed to be 1.10 in urban areas and 1.05 in rural areas. The household response rate was fixed at 96 per cent for both urban and rural areas and the women’s individual response rate was 98 per cent for both urban and rural areas. The objectives of the 2011 BDHS is to provide data on knowledge and attitudes of women about sexually transmitted infections and HIV/AIDS.

### Data analysis

Statistical analysis of the results was obtained by using Statistical Packages Program for Social Sciences (SPSS-21) for Windows Version 21. Bivariate analyses for the variables were presented in percentage form. The χ2 tests were used to determine the significant association with the dependent variable. Logistic regression analysis was conducted to determine the risk factors with the dependent variable. A two-tailed p value of 0.05 was considered significant at the 95% CI (Confidence Interval) level. Logistic regression analysis was also conducted to determine the risk factors with the dependent variable. The dependent variable used in the model was a dichotomous binary variable: Y = 1, if the respondents have knowledge and awareness about STDs and y = 0, otherwise. Division, places of residence, respondent’s age, education, radio and watch TV were considered as explanatory variables in this model.

### Ethics statement

Ethical approval was obtained from the Ministry of Health and Family Welfare, Dhaka, Bangladesh. International credit finance (ICF) provided financial and technical assistance for the survey through USAID/Bangladesh. The BDHS is part of the worldwide Demographic and Health Surveys programme, which is designed to collect data on fertility, family planning, and maternal and child health. Informed written consent was obtained from all participants prior to the study.

## Results

The results of the association between knowledge and awareness about STDs among the selected socio-demographic characteristics of women in Bangladesh are presented in Table 1. Table 1 reveals that most (70.6%) of the respondents have knowledge and awareness about STDs, of which 9.0%, 16.4%, 12.5%, 12.5%, 8.1% and 12.1% represent the results from Barisal, Dhaka, Khulna, Rajshahi, Sylhet and Chittagong division, respectively. Therefore, the knowledge and awareness about STDs among the people in Dhaka is higher than that of the others divisions, while the Sylhet division shows the lowest percentage. In Bangladesh, the majority (70.6%) of women have knowledge and awareness about STDs, while 29.4% of them do not have any knowledge or awareness about STDs. The Dhaka division has the highest percentage of respondents with knowledge and awareness about STDs (16.4%). This could be due to the Dhaka division being in Bangladesh’s capital city, which is where most of the educated people live. Among all the respondents, 62.2% and 37.8% live in rural and urban areas, respectively, where 38.2% and 32.2%, respectively, have knowledge and awareness about STDs. It is clear that the knowledge and awareness about STDs among the urban population is greater than that of the rural population. In Bangladesh 29.7%, 30.4% and 7.9% of women have completed primary, secondary and higher level of education, respectively, of which 20.3%, 27.9% and 7.8%, respectively, know about STDs. However, about one-third of the respondents are illiterate with only 14.7% knowing about STDs while less than one-quarter do not know. Therefore, it may be concluded that the knowledge and awareness about STDs increases with education. Again, 12.3%, 37.4% and 50.4% respondents belong to the 15-19, 20-29 and 30-49 years age groups, respectively, of which 9.6%, 29% and 32% have knowledge and awareness about STDs, respectively. It is also observed that the younger aged populations have more knowledge and awareness about STDs while the populations aged 30-49 years have less knowledge and awareness. A total of 68.2% of the women respondents do not know about STDs, while 31.8% of respondents know about STDs from listening to the radio and 42.6% of the respondents know about STDs from watching television (TV). As radio and TV play an important role in increasing knowledge and awareness about STDs and HIV/AIDS, more effective and special programmes should be broadcast on the radio and TV. This may help to increase the knowledge and awareness about STDs. The respondent’s age, education, place of residence, division, listening to the radio and watching TV is found to be significantly associated with knowledge and awareness among women in Bangladesh about STDs (Table 1).

**Table 1:** **The results of Chi-square (χ** ^**2**^ **) test of knowledge and awareness about STDs among various socio-economic and demographic variables**

Variables		Frequency (Percentage)	Knowledge and awareness about STDs			χ ^2^ values
			No (%)	Yes (%)	Total (%)	
**Division**	Barisal	2066(13.8)	452 (4.1)	986 (9.0)	1438 (13.1)	240.57^*^
	Dhaka	2284(16.0)	534 (4.9)	1806 (16.4)	2340 (21.3)	
	Khulna	2656(24.2)	332 (3.0)	1379 (12.5)	1711 (15.6)	
	Rajshahi	1319(12.9)	704 (6.4)	1376 (12.5)	2080 (18.9)	
	Sylhet	1120(10.2)	595 (5.4)	889 (8.1)	1484 (13.5)	
	Chittagong	2871(22.9)	616 (5.6)	1327 (12.1)	1943 (17.7)	
**Place of residence**	Urban	4106(37.3)	585 (5.3)	3566 (32.4)	4151 (37.8)	752.87^*^
	Rural	6890(62.7)	2648 (24.1)	4197 (38.2)	6845(62.2)	
**Respondent’s education**	Illiterate	2505(22.8)	1910 (17.4)	1615 (14.7)	3525 (32.1)	2115.35^*^
	Primary	3311(30.1)	1041 (9.5)	2227 (20.3)	3268 (29.7)	
	Secondary	4194(38.1)	280 (2.5)	3065 (27.9)	3345 (30.4)	
	Higher	986(9.0)	2 (0.1)	853 (7.8)	855 (7.9)	
**Respondent’s age**	15-19 yrs	3425(31.1)	296 (2.7)	1052 (9.6)	1348 (12.3)	268.61^*^
	20-29 yrs	5124(46.6)	917 (8.3)	3192 (29.0)	4109 (37.4)	
	30-49 yrs	2447(22.3)	2020 (18.4)	3519 (32.0)	5539 (50.4)	
**Radio**	No	943(8.6)	2529 (23.0)	4967 (45.2)	7496 (68.2)	213.34^*^
	Yes	10053(91.4)	704 (6.4)	2796 (25.4)	3500 (31.8)	
**Watch TV**	No	6187(56.3)	2665 (24.2)	3646 (33.2)	6311 (57.4)	1173.97^*^
	Yes	4809(43.7)	568 (5.2)	4117 (37.4)	4685 (42.6)	

Notes: ^*^p value < 0.001.

Table 2 represents the results of the logistic regression model. According to the fitted model, all of the selected variables appear as significant predictors in the case of knowledge and awareness among women in Bangladesh about STDs. In accordance with their importance, respondent’s age, education, place of residence, division, listening to radio and watching TV have a statistically significant effect on the knowledge and awareness among women in Bangladesh about STDs. Based on this analysis, it is found that the respondent’s current age is the most significant factor affecting the knowledge and awareness of women about STDs. The results also show that women who are aged 20-29 and 30-49 years have 1.331 times more knowledge and awareness, and 0.978 times less knowledge and awareness about STDs, respectively, than that of the respondents who are aged 15-19. This shows that women aged 20-29 years have greater knowledge and awareness about STDs than women of other ages.

**Table 2:** **The results of the logistics analysis of knowledge and awareness about STDs among various socio-economic and demographic variables**

Variables		Co-efficient (β)	S.E	df	P value	Exp(β)	95% CI
**Division**	Barisal (rc)			4	0.000	1.000	
	Dhaka	0.512	0.077	1	0.000	1.669	0.89-2.1
	Khulna	0.804	0.078	1	0.000	2.234	1.2-3.2
	Rajshahi	-0.052	0.082	1	0.547	0.943	0.58-1.67
	Sylhet	-0.067	0.155	1	0.467	0.949	0.54-1.30
	Chittagong	-0.029	0.062	1	0.742	0.971	0.87-1.10
**Place of residence**	Urban (rc)	…….	……	1	0.000	1.000	
	Rural	-1.020	0.062	1	0.000	0.363	0.20-1.08
**Respondent’s education**	No-education (rc)	…….	……	3	0.000	1.000	
		0.861	0.060	1	0.000	2.366	1.98-3.1
	Primary	2.311	0.078	1	0.000	10.089	8.98-12.77
	Secondary Higher	5.314	0.710	1	0.000	20.241	18.33-22.65
**Respondent’s age**	15-19 (rc)			2	0.000	1.000	
	20-29	0.286	0.066	1	0.001	1.331	0.98-2.31
	30-49	-0.022	0.076	1	0.800	0.978	0.88-1.70
**Radio**	No (rc)			1	0.000	1.000	
	Yes	0.173	0.108	1	0.004	1.189	1.29-3.12
**Watch TV**	No (rc)	…..	……	1	0.000	1.000	
	Yes	0.916	0.062	1	0.000	2.498	2.22-4.09

Model Summary:

2 Log likelihood: 9217.670.

Cox & Snell R Square: 0.56.

Negelikereke R: 0.66.

Model Chi-Square: 2915.402.

Notes: rc = reference category, S.E = Standardization Error and df = degree of freedom.

Women’s education is also an imperative factor for knowledge and awareness about STDs. The regression coefficients for women who have completed primary, secondary and higher levels of education have 2.366, 10.089 and 20.241 times greater knowledge and awareness about STDs than women who have no education, respectively. This shows that the level of knowledge and awareness about STDs among women increases with the increasing level of education. The odds ratio for women living in rural areas is 0.363, which shows that, rural women have 0.363 times less knowledge and awareness about STDs than the women who live in urban areas. This could be because women in rural areas have access to poorer facilities than women in urban areas, which is essential for increasing knowledge and awareness about STDs.

The regression odds ratios for women living in Dhaka, Khulna, Rajshahi, Sylhet and Chittagong are 1.669, 2.234, 0.943, 0.949 and 0.971, respectively. These results show that women living in Dhaka and Khulna have 1.669 and 2.234 times more knowledge and awareness about STDs than Barisal, Rajshahi, Sylhet and Chittagong divisions. Rajshahi, Sylhet and Chittagong divisions have 0.943, 0.949 and 0.971 times less knowledge and awareness about STDs than the women living in the divisions of Dhaka, Khulna and Barisal in Bangladesh. Since the women of Rajshahi, Sylhet and Chittagong division have less knowledge and awareness about STDs than women from the other divisions, and have more risk of STDs, it is essential to provide more programmes to increase their knowledge and awareness about STDs. The regression coefficient of the women who listened to the radio is 1.189, which indicates that the women who listen to the radio have 1.189 times more knowledge and awareness about STDs than the women who do not listen to the radio. The regression coefficients of women who watch TV is 2.498, which shows that women who watch TV have 2.498 times more knowledge and awareness about STDs than women who do not watch TV.

For the fitted model, the Cox and Snell *R*^2^ = 0.56 and Nagelkerke . It is observed that when the value of  exceeds 0.5 the data fit the binary logistic regression model well. Therefore, the model can be used for the prediction of significance about the knowledge and awareness among women in Bangladesh about STDs.

## Discussion

The various socio-economic and demographic factors play a crucial role in influencing STDs in Bangladesh. This study was designed to determine the knowledge and awareness about STDs among women who are a vulnerable group based on the global threat that STDs present to humankind. However, it is difficult in a poor setting like Bangladesh, where proper steps need to be taken to improve the situation of education in rural areas as well as throughout the country. However, there is a real need for more in-depth studies on this topic. Our aim was to explore the knowledge and awareness of STDs among women, who are a neglected section of the society, concerning access to information and services.

Among the respondents of this study, the majority were aged 15 to 29 years of age, which is in agreement with some studies [16–19]. Women of increasing age are exposed to new experiences relating to sexuality and reproduction [16]. Interestingly, the 15-29 years age group have relatively good knowledge and awareness about STDs, and the association is statistically significant (p < 0.001). Such a degree of poor knowledge and awareness in the younger group (<29 year) may be due to the fact that the traditional social system and health care service often ignore this group as a separate entity who are treated neither as a child nor as an adult. Their age remains the main barrier to communication with adults regarding information concerning sex and sexually transmitted diseases. Thus, necessary action is required to reduce the level of STDs in the country in order to achieve better living conditions in the future [20].

There is a significant association between the knowledge and awareness about STDs with the level of education of women. Education positively contributes to the knowledge and awareness on STDs, which is also statistically significant in this study (p < 0.000). In fact, education is the pathway of communication for any message. The increased age with the increased level of education provides an opportunity to have more reproductive health information, as well as more use of heath care services and support from peer groups [16]. This is in agreement with this study [21]. It is found that AIDS awareness is much greater among students of St. Mary’s college than other students in Meghalaya. This reflects the positive influence of literacy for the improvement of knowledge [16, 17] and [22] the similar associations with this study. This can be explained by the fact that educated people can acquire more knowledge when they are exposed to sources of available information like electronic media (computer, Internet) and printed paper (books, newspapers, posters, booklets).

Women with access to mass media have the opportunity to share their views and ideas with their friends and so they are more aware of the fact. In this study, almost all the respondents said that they have heard about STDs. This is very similar to a study in Tanzania [19, 23]. In comparing the knowledge and awareness about STDs, it showed that 17% of adolescents in Bangladesh had never heard of STDs; Indians and Nepalese reported 37% and 25%, respectively [16]. His study reflects the general adolescent population of these countries, unlike our study, which concentrates on a sample population in Dhaka city who are provided with more facilities to obtain information from the mass media. Mass media exposure, such as radio and TV, is positively associated with having knowledge and awareness about STDs among the adolescents. The exposure to such media can communicate knowledge and awareness about STDs through music, news reports, songs, dramas, documentaries and advertising, and can profoundly influence the attitude and behaviour of people [16]. The considerable contribution of the mass media was shown in a mass media project using a TV programme to provide adolescents in Zaire with knowledge and awareness about STDs [21]. This is similar to the association found by Khan and Goel in their study [16, 19]. The major source of information about STDs for our respondents is the radio and TV. One of the limitations in this study is that the information was self-reported.

## Conclusion

The knowledge and awareness about STDs in Bangladesh has been a topic of interest in population research because of its apparent direct relationship with the lack of health facilities, and, indirectly, with poverty. Women’s education is also an imperative factor for knowledge and awareness about STDs. This could be because women in urban areas have access to better facilities than women in rural areas, which is essential for increasing knowledge and awareness about STDs. The women from the Rajshahi, Sylhet and Chittagong divisions have less knowledge and awareness about STDs than women from the other divisions and have more risk of STDs. It is essential to provide more programmes to increase their knowledge and awareness about STDs. However, the radio and TV play an important role in increasing the knowledge and awareness about STDs as well as HIV/AIDS. Hence, special programmes can increase knowledge and awareness about STDs in Bangladesh and should be broadcast on the radio and television networks. As the level of knowledge and awareness on STDs is found to increase with age and literacy, educational intervention programmes, may also be effective for women in Bangladesh.
